# Application of Diffusion Kurtosis Imaging and Blood Oxygen Level-Dependent Magnetic Resonance Imaging in Kidney Injury Associated with ANCA-Associated Vasculitis

**DOI:** 10.3390/tomography10070073

**Published:** 2024-06-25

**Authors:** Wenhui Yu, Weijie Yan, Jing Yi, Lu Cheng, Peiyi Luo, Jiayu Sun, Shenju Gou, Ping Fu

**Affiliations:** 1 Division of Nephrology, West China Hospital of Sichuan University, Chengdu 610041, China; yuwenhui1@stu.scu.edu.cn (W.Y.); yijing202406@163.com (J.Y.); chenglu202406@163.com (L.C.); l178011870@163.com (P.L.); fuping@scu.edu.cn (P.F.); 2 Division of Radiology, West China Hospital of Sichuan University, Chengdu 610041, China; yanweijie@wchscu.cn (W.Y.); sunjiayu@wchscu.cn (J.S.); 3 Division of Nephrology, West China Airport Hospital of Sichuan University, Chengdu 610200, China

**Keywords:** ANCA-associated vasculitis, magnetic resonance imaging, diffusion magnetic resonance imaging, kurtosis

## Abstract

Objective: Functional magnetic resonance imaging (fMRI) has been applied to assess the microstructure of the kidney. However, it is not clear whether fMRI could be used in the field of kidney injury in patients with Antineutrophil cytoplasmic antibody (ANCA)-associated vasculitis (AAV). Methods: This study included 20 patients with AAV. Diffusion kurtosis imaging (DKI) and blood oxygen level-dependent (BOLD) scanning of the kidneys were performed in AAV patients and healthy controls. The mean kurtosis (MK), mean diffusivity (MD), and fractional anisotropy (FA) parameters of DKI, the R2* parameter of BOLD, and clinical data were further analyzed. Results: In AAV patients, the cortex exhibited lower MD but higher R2* values compared to the healthy controls. Medullary MK values were elevated in AAV patients. Renal medullary MK values showed a positive correlation with serum creatinine levels and negative correlations with hemoglobin levels and estimated glomerular filtration rate. To assess renal injury in AAV patients, AUC values for MK, MD, FA, and R2* in the cortex were 0.66, 0.67, 0.57, and 0.55, respectively, and those in the medulla were 0.81, 0.77, 0.61, and 0.53, respectively. Conclusions: Significant differences in DKI and BOLD MRI parameters were observed between AAV patients with kidney injuries and the healthy controls. The medullary MK value in DKI may be a noninvasive marker for assessing the severity of kidney injury in AAV patients.

## 1. Introduction

Antineutrophil cytoplasmic antibody (ANCA)-associated vasculitis (AAV) is a group of autoimmune diseases involving various systems of the body [1]. Kidney involvement is common in patients with AAV and typically presents as a rapidly progressive deterioration of renal function. Kidney involvement is an important predictor of mortality in AAV [2]. Renal histology, which indicates the microstructure of the kidney, is an important reference for the diagnosis, treatment, and prognosis assessment of patients with AAV [3,4]. However, performing repeated renal biopsies to assess the dynamic changes in kidney injury may be challenging owing to the invasive nature and associated potential complications. Most patients with AAV are older adults, which increases the risk of complications from kidney biopsy, such as bleeding. In addition, sampling error is another limitation of kidney biopsies. Therefore, a noninvasive and reproducible method is required to evaluate kidney injury in patients with AAV.

Functional magnetic resonance imaging (fMRI), a newly developed noninvasive technique with quantitative imaging parameters, has been applied to assess the microstructure and function of the kidney in different diseases [5]. Scanning the whole kidney using fMRI can avoid the sampling bias caused by renal biopsy. Diffusion kurtosis imaging (DKI) MRI has been applied to evaluate the microstructure of animal and human tissues, specifically the fibrosis change, based on the principle of non-Gaussian diffusion of water molecules [6,7,8,9], Blood oxygen level-dependent (BOLD) MRI could assess tissue oxygenation by using the paramagnetic properties of deoxyhemoglobin. The apparent relaxation rate R2* value of BOLD-MRI is correlated with the local deoxyhemoglobin levels. BOLD-MRI is sensitive to intrarenal oxygen availability and helps predict the progressive decline in renal function in patients with kidney disease, such as chronic kidney disease, acute allograft dysfunction, and lupus nephritis [10,11,12,13,14]. In patients with AAV, the percentage of sclerotic glomeruli and chronic tubulointerstitial lesions, such as fibrosis in renal histopathology, are strong predictors of poor renal outcomes [15]. Theoretically, fMRI, such as DKI and BOLD, which can reflect fibrosis or ischemic lesions in the kidney microstructure, might be applied prospectively in AAV. However, to our knowledge, no published studies have revealed the use of DKI or BOLD MRI for kidney injury in patients with AAV. Hence, this study aimed to explore the value of DKI and BOLD MRI in assessing the severity and prognosis of kidney injury in patients with AAV.

## 2. Methods

### 2.1. Study Population

This study included 20 patients who had been diagnosed with AAV at the West China Hospital of Sichuan University between November 2018 and April 2021. The diagnosis of AAV was made according to the criteria of the 2012 Chapel Hill Consensus Conference [16]. Patients who met the following criteria were excluded: (1) continuous dialysis for >3 months; (2) inability to cooperate because of objective reasons, such as deafness, pregnancy, or mental disorder; (3) presence of a large amount of ascites, solitary kidney, or multiple kidney stones; (4) contraindications for MRI examination; (5) age < 18 years; and (6) presence of concomitant diseases, such as lupus nephritis and diabetic nephropathy. The sample size was calculated according to the formula used to calculate the sample size in a cross-sectional clinical diagnostic test. Clinical data were obtained from the Hospital Information System (HIS), and the estimated glomerular filtration rate (eGFR) was calculated using the Chronic Kidney Disease Epidemiology Collaboration (CKD-EPI) equation. The renal histology of the patients was evaluated according to a previous protocol [17,18,19]. The percentages of normal glomeruli in the total number of glomeruli, the percentages of glomeruli with crescents, the percentages of glomeruli with cellular crescents, the percentages of glomeruli with cell fibrous crescents, the percentage of the tubulointerstitial compartment with fibrosis in the whole tubulointerstitial compartment, and the percentage of the tubulointerstitial compartment with tubular atrophy in the whole tubulointerstitial compartment were recorded. All patients were followed for six months. The life and kidney living state were recorded. Endpoint events were defined as maintenance dialysis for more than three months, an increase in serum creatinine >30% at baseline, or death.

The control group comprised 15 sex- and age-matched healthy volunteers. The specific inclusion criteria were as follows: (1) no history of cardiovascular disease, diabetes, or kidney disease, and no medication intake exceeding >15 days that may affect the physiological status of the kidneys, (2) aged between 18 and 80 years, and (3) no contraindications to the MRI examination.

This study was approved by the Institutional Ethics Review Committee of the West China Hospital of Sichuan University (approval number: 2019964). Written informed consent was obtained from all the participants.

### 2.2. fMRI Protocol

DKI (diffusion kurtosis imaging) and BOLD (blood oxygen level-dependent) are two significant functional magnetic resonance imaging (fMRI) techniques commonly used in the study and diagnosis of pathological changes in various tissues and organs. DKI can provide a detailed description of the complex diffusion behavior of water molecules within tissues by measuring the non-Gaussian diffusion of these molecules. BOLD imaging is based on changes in blood oxygenation levels, relying on the magnetic differences between oxygenated and deoxygenated hemoglobin to generate contrast. This technique is used to assess tissue oxygenation status. In renal research, BOLD can be utilized to detect oxygen supply and consumption in kidney tissues.

fMRI was performed using a GE 3.0T Discovery MR750W superconductor scanner equipped with a 16-channel body coil. The magnetic field gradient was 44 mT/m, and the magnetic field switching rate was 200 mT/ms. All participants were instructed to observe fasting and refrain from drinking water for at least 6–8 h before the examination to reduce the impact of intestinal contents on image quality. Prior to the MRI scan, all participants were informed of the precautions and adequately trained regarding the breathing practice: slow and deep inhalation, quick exhalation, and holding their breath for at least 15 s each time. The coronal T1-weighted sequence and coronal T2-weighted sequence were obtained. The coronal BOLD sequence required the participants to hold their breath for at least 15 s, whereas the coronal DKI sequence required the participants to breathe freely. BOLD MRI was performed using a GE 3.0T system (GE Medical Systems, Discovery MR 750w). BOLD images of coronal slices were acquired through the renal hilum during breath-holding using a multiple gradient echo sequence. The BOLD-MRI parameters were as follows: echoes, 10; repetition time (TR), 33.6 ms; echo time (TE), 1.9–37.1 ms; flip angle, 25°; slice thickness, 4 mm; interval, 0 mm; field of view, 30 × 30 cm^2^; matrix, 96 × 96; and number of excitations (NEX) of 1. DKI-MRI was performed using a GE 3.0T system. DKI was performed using a free-breathing single-shot echo-planar sequence in the coronal plane with the following parameters: 7500 ms TR, 66 ms TE, 4 mm section thickness, 30 × 30 cm field of view, and 96 × 96 matrix. The b values were 0, 500, and 1000 s/mm^2^ with NEX numbers of 3, 3, and 3, respectively, and were applied in three diffusion directions. The diffusion scheme used was monopolar. The acquisition time of DKI was 5 min and 40 s.

### 2.3. Image Analysis

**Figure 1:**
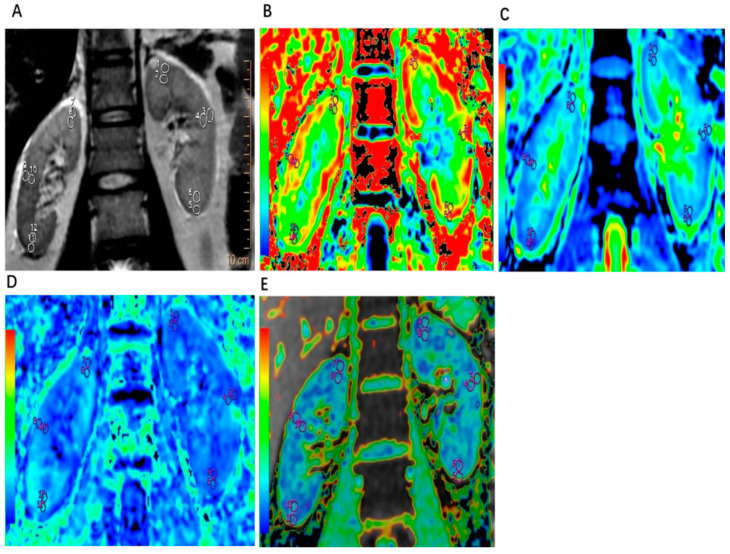
Drawing the regions of interest (ROIs) illustration. In the images, 1, 3, and 5 indicate the ROIs of the upper, middle, and lower poles of the left kidney cortex, respectively; and 7, 9, and 11 indicate the ROIs of the upper, middle, and lower poles of the right kidney, respectively. In addition, 2, 4, and 6 indicate the ROIs of the upper, middle, and lower poles of the left renal medulla, respectively, and 8, 10, and 12 indicate the ROIs of the upper, middle, and lower poles of the right renal medulla, respectively. (**A**). Example of drawing ROIs on T2 MRI results of a kidney from a patient with AAV. (**B**). Example of drawing ROIs on DKI-MK scanning results of a kidney from a patient with AAV. (**C**). Example of drawing ROIs on DKI-MD scanning results of a kidney from a patient with AAV. (**D**). Example of drawing ROIs on DKI-FA scanning results of a kidney from a patient with AAV. (**E**). Example of drawing ROIs on BOLD-R2* scanning results of a kidney from a patient with AAV.

The original image was processed on the Funtool software platform (adw4.6). The placement of regions of interest (ROIs) was performed independently by two radiologists with more than 5 years of experience in abdominal imaging diagnosis. T1-weighted MRI images were used as anatomical reference images to distinguish the cortex and medulla. On delineating ROIs on T1-weighted MRI images, the system automatically calculated the image parameter values (DKI sequence: MK, MD, FA; BOLD sequence: R2*). The values of MK, MD, FA, and R2* were recorded. The measurements were conducted two times, and the average values of both kidneys were taken as the final recorded data. ROIs were oval-shaped, and the areas of ROIs were between 20 and 40 mm^2^. Three ROIs were drawn on each side of the renal cortex and medulla, which were evenly distributed in the upper pole, hilum plane, and lower pole of the kidney. Figure 1 illustrates the ROI drawing method.

### 2.4. Statistical Analysis

All statistical analyses were performed using SPSS 24.0 software (IBM, Corp. Armonk, NY, USA). Data are expressed as the means ± standard deviation for normally distributed quantitative parameters, and a *t*-test or Pearson correlation analysis was used for data analysis. Non-normally distributed data are expressed as the median (interquartile range) and analyzed using the Mann–Whitney U test or Spearman correlation analysis. Receiver operating characteristic (ROC) curve analysis was performed to assess the diagnostic performance and determine the optimal cutoff value of each diffusion parameter for predicting the degree of renal injury. The areas under the curve (AUCs) were calculated using the DeLong method. Statistical significance was set at *p* < 0.05.

## 3. Results

### 3.1. Demographic and General Data of Patients with AAV

Among 20 patients with active AAV, 12 were women and 8 were men, with a mean age of 55.8 ± 11.5 years at diagnosis. The mean level of serum creatinine was 366.0 ± 203.9 μmol/L, and the mean Birmingham Vasculitis Activity Score (BVAS) was 19.0 ± 4.1. BOLD MRI scanning was completed in all patients, and DKI scanning was completed in 19 patients. Eight patients underwent renal biopsy during hospitalization, and histopathological information was collected. Seven among them underwent DKI scanning. Table 1 summarizes the general clinical data of patients with AAV collected within three days of fMRI scanning.

### 3.2. Comparison of Imaging Parameters of DKI and BOLD MRI between the Renal Cortex and Medulla

**Figure 2:**
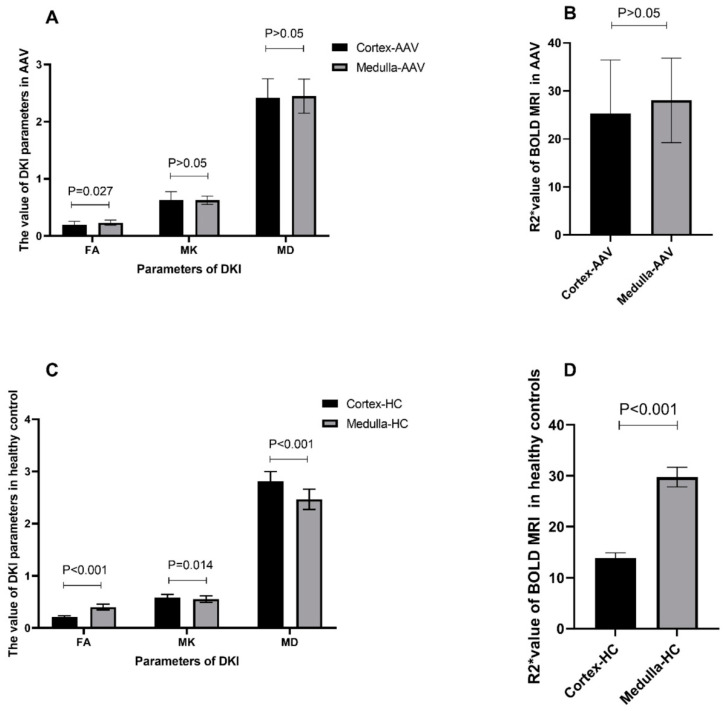
Comparison of DKI and BOLD MRI parameters between the cortex and medulla in the kidneys of patients with AAV and the healthy controls (HCs). (**A**) Comparison of DKI MRI parameters (FA, MK, and MD values) between the cortex and medulla in the kidneys of patients with AAV. (**B**) Comparison of BOLD MRI parameters (R2*values) between the cortex and medulla in the kidneys of patients with AAV. (**C**) Comparison of DKI MRI parameters (FA, MK, and MD values) between the cortex and medulla in the kidneys of HCs. (**D**) Comparison of BOLD MRI parameters (R2*value) between the cortex and medulla in the kidneys of HCs.

MK, MD, and FA parameters were recorded for the DKI sequence, and R2* parameters were recorded for the BOLD MRI sequence. The FA value of the cortex was lower than that of the medulla in the AAV group (0.197 ± 0.059 vs. 0.232 ± 0.046, *p* = 0.027). No significant differences were observed in the MD, MK, and R2* values of the cortex and medulla in the AAV group (Figure 2A,B).

The FA value of the cortex was lower than that of the medulla in the healthy control group (0.21 ± 0.025 vs. 0.403 ± 0.055, *p* < 0.001), the MK value of the cortex was higher than that of the medulla (0.585 ± 0.059 vs. 0.553 ± 0.063, *p* = 0.014), the MD value of the cortex was higher than that of the medulla (2.812 ± 0.186 vs. 2.464 ± 0.195, *p <* 0.001), and the R2* value of the cortex was lower than that of the medulla (13.832 ± 0.999 vs. 29.73 ± 1.933, *p* < 0.001) (Figure 2C,D).

### 3.3. Comparison of fMRI Parameters between Patients with AAV and Healthy Controls

**Figure 3:**
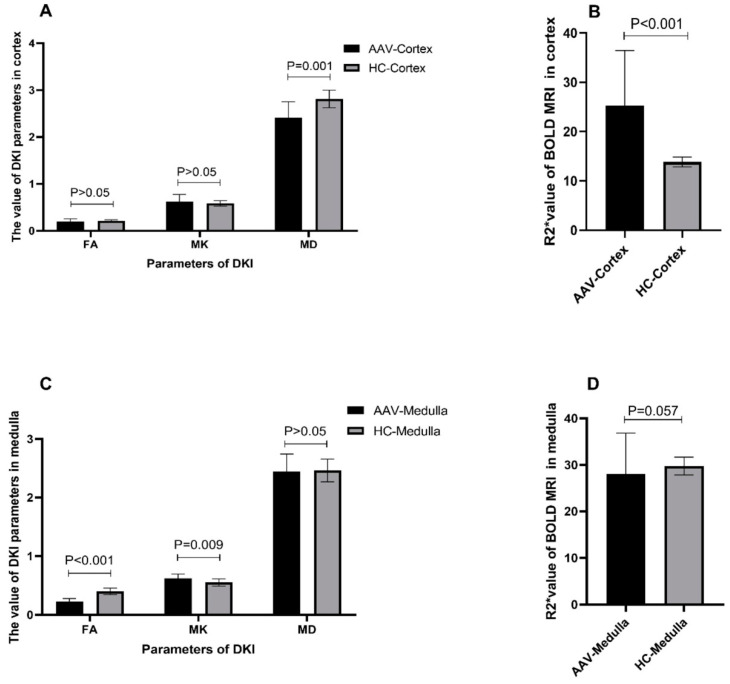
Comparison of DKI and BOLD MRI parameters in the kidney cortex and medulla between patients with AAV and the healthy controls (HCs). (**A**). Comparison of DKI MRI parameters (FA, MK, and MD values) in the kidney cortex between AAV and HC patients. (**B**). Comparison of BOLD MRI parameters (R2*value) in the kidney cortex of AAV and HC patients. (**C**). Comparison of DKI MRI parameters (FA, MK, and MD values) in the kidney medulla between AAV patients and HCs. (**D**). Comparison of BOLD MRI parameters (R2*values) in the kidney medulla between patients with AAV and HCs.

The R2* value of the cortex in patients with AAV was higher than that in the healthy controls (25.241 ± 11.19 vs. 13.832 ± 0.999, *p* < 0.001). The MD value of the cortex in patients with AAV was lower than that in the healthy controls (2.416 ± 0.334 vs. 2.812 ± 0.186, *p* = 0.001), whereas no significant differences in the FA and MK values in the cortex were observed between patients with AAV and the healthy controls (Figure 3A,B). The FA value of the medulla was lower in patients with AAV than in the healthy controls (0.232 ± 0.046 vs. 0.403 ± 0.055, *p* < 0.001). The MK value of the medulla was higher in patients with AAV than in the healthy controls (0.626 ± 0.074 vs. 0.553 ± 0.063, *p* = 0.009), whereas no significant difference in the MD and R2* values in the medulla were observed between patients with AAV and the healthy controls (Figure 3C,D).

### 3.4. Association between fMRI Parameters and Clinicopathological Parameters of Patients with AAV

Of the 20 patients with AAV, correlation analysis showed that the MK value of the renal medulla was correlated with the serum creatinine level (r = 0.542, *p* = 0.017). The MK value of the renal medulla was inversely correlated with the hemoglobin level and eGFR (r = −0.0457, *p* = 0.049; r = −0.539, *p* = 0.017, respectively). The MK value of the renal cortex was negatively correlated with hemoglobin level (r = −0.502, *p* = 0.029). The R2* value of the renal medulla correlated with the age of the patients (r = 0.469, *p* = 0.037). The correlation analysis of eight patients who had undergone renal biopsy showed that the MK value of the renal cortex was correlated with the percentage of crescents (r = 0.927, *p* = 0.01), whereas the MK value of the renal medulla was not correlated with the percentage of crescents (r = 0.741, *p* = 0.07) (Table 2).

### 3.5. ROC Analysis of Diffusion Parameters

Table 3 shows the ROC analysis results for multiple diffusion parameters predicting the degree of kidney injury. The AUC values of MK, MD, FA, and R2* in the cortex were 0.57, 0.87, 0.60, and 0.95, respectively, whereas the AUC values of MK, MD, FA, and R2* in the medulla were 0.77, 0.54, 0.99, and 0.69, respectively, for discrimination between the healthy controls and AAV patients. The medullary FA achieved the largest AUC, suggesting that the medullary FA may be the best parameter for discriminating kidney injuries between AAV patients and the healthy controls. Moreover, the sensitivity, specificity, and optimal cutoff values were 94.7%, 100%, and 0.29, respectively. In patients with AAV, a creatinine level ≥300 µmol/L was defined as severe kidney injury, and ≤300 µmol/L was defined as mild kidney injury. To differentiate the degree of renal injury in AAV patients, the AUC values of MK, MD, FA, and R2* in the cortex were 0.66, 0.67, 0.57, and 0.55, respectively, while the AUC values of MK, MD, FA, and R2* in the medulla were 0.81, 0.77, 0.61, and 0.53, respectively. The medullary MK achieved the largest AUC, indicating that it may be the best parameter for discriminating the severity of renal injury in AAV patients. The sensitivity, specificity, and optimal cutoff values were 81.8%, 75%, and 0.59, respectively (Table 3).

### 3.6. Follow-Up of Patients with AAV

Of the 20 patients, 9 patients with AAV reached the endpoint event at the six-month follow-up, including maintenance dialysis for more than three months in five patients. The serum creatinine of one patient increased by >30% at baseline, and one patient was reported dead and another patient was lost to follow-up. No significant difference in the values of fMRI parameters of the kidney were observed between patients with AAV who reached the endpoint event and those who did not (Table 1).

## 4. Discussion

As newly developed noninvasive imaging techniques, DKI and BOLD MRI have been applied to a variety of kidney diseases. However, whether DKI and BOLD MRI are applicable for patients with AAV is unknown. Therefore, in the present study, we performed DKI and BOLD MRI scanning in patients with AAV and in the healthy controls to investigate changes in fMRI parameters in AAV patients and the correlation between imaging and clinical parameters.

In the present study, the MK value in the renal medulla of AAV patients was higher than that in the healthy controls. As a kurtosis parameter in DKI, MK has been hypothesized to quantify the deviation in the actual water molecule distribution from the ideal water molecule distribution [20]. An increased MK value suggests that the tissue has an irregular and complex environment. The renal histopathology of patients with AAV revealed localized vessel wall necrosis, proliferative crescent formation, tubular atrophy, interstitial fibrosis, and various types of inflammatory cell infiltration [21]. All of the above changes could lead to more irregular and heterogeneous microstructures than those in normal kidneys, leading to an increase in MK values. Our results are consistent with those of previous studies on diabetic nephropathy and chronic kidney disease, which also showed an increase in the medullary MK value in the renal disease group compared with the healthy control group [22,23]. A similar change in the MK value of the renal medulla caused by different kidney diseases may reflect a certain consistency in imaging manifestations. The medullary MK value in patients with AAV correlated with serum creatinine levels and negatively correlated with the eGFR, indicating that the medullary MK value correlated well with the severity of kidney injury. In addition, when classifying the severity of the patient’s renal function based on a threshold of 300 μmol/L of creatinine, the medullary MK value achieved the largest AUC, suggesting that it may be the best parameter to discriminate the severity of kidney injury in AAV patients using ROC analysis. The sensitivity and specificity were 81.82% and 75%, respectively, which are consistent with the ROC analysis data of fMRI in distinguishing the severity of kidney injury in patients with chronic kidney disease [23]. The MK value reflects the degree to which water molecules deviate from the Gaussian distribution [24,25], which is related to the properties of the tissue microstructure. The larger the MK value, the more complex the renal microstructure and the more serious the renal lesions. As shown in the present study, the MK values of the renal medulla correlated with the severity of renal damage, indicating that the microstructure of the kidney becomes increasingly complex with the progression of ANCA disease. Therefore, the MK value of DKI MRI scanning may be a potential noninvasive biomarker of renal injury severity in patients with AAV.

Consistent with the findings of previous studies, the MD value of the renal cortex in this study was higher than that of the medulla in the healthy controls [20]. The MD of the renal cortex in AAV patients was significantly lower than that in the healthy controls, suggesting that the MD of the renal cortex decreased in AAV patients with renal injury. As a parameter for DKI scanning that analyzes non-Gaussian water diffusivity, the MD value reflects the diffusion coefficient corrected by a non-Gaussian bias [25]. Decreased MD values usually suggest that the tissue contains more components that limit the diffusion of water molecules. For instance, in tumors, cell proliferation increases cell density and decreases extracellular space, restricting the diffusion of water molecules and decreasing the MD value [26]. In the pathogenesis of AAV, glomerulosclerosis, crescent formation, renal interstitial fibrosis, extracellular matrix deposition, and inflammatory cell infiltration can increase barriers to the diffusion of water molecules. These pathological changes in the kidney cortex may contribute to a decrease in the cortical MD value in patients with AAV. The FA value of the renal cortex in the healthy controls was significantly lower than that of the medulla, which is consistent with most previous studies [20,27]. FA values have been hypothesized to represent the direction of water mobility [28]. A decreased FA value indicates that the tissue has less structure to form water-molecule anisotropy. In healthy kidneys, the radial orientation of tubules that were mostly distributed in the kidney medulla rather than in the kidney cortex restricted the movement of water molecules in the medulla more than in the cortex. Consequently, the FA value was higher in the medulla than in the cortex [29]. The FA value of the renal medulla in patients with AAV was significantly lower than that in the healthy controls, suggesting that the FA value of the renal medulla was decreased in patients with AAV. Tubular atrophy, interstitial inflammation, and fibrosis are common histologic manifestations of kidney injury in AAV patients [21]. Under these conditions, the pipeline system in the medulla, which forms water molecule anisotropy, is destroyed, resulting in a decrease in the FA value in the medulla of patients with AAV. Similar results have been obtained in diabetic nephropathy, lupus nephropathy, and obstructive nephropathy [30,31].

Furthermore, the R2* value of the renal cortex was lower than that of the medulla in the healthy controls, which is consistent with previous studies [32,33,34]. As a characteristic index of BOLD MRI scanning, R2* has been hypothesized to indicate tissue oxygenation: a high R2∗ value suggests low tissue oxygenation, and vice versa [35]. Under physiological conditions, the oxygen partial pressure in the renal cortex is higher than that in the medulla, which may have contributed to the lower R2* values in the renal cortex. In the present study, the R2* value of the renal cortex in patients with AAV was much higher than in the healthy controls, suggesting that the R2* value of the renal cortex increased in patients with AAV. Renal oxygenation was determined based on the delivery and consumption of oxygen. In terms of oxygen supply, the main histologic characteristics of AAV are the presence of necrotizing inflammation and fibrinoid necrosis in the walls of small- and medium-sized vessels [36], especially in the glomerular loops, which are distributed mainly in the renal cortex. The destruction of blood vessels causes a reduction in blood supply, resulting in kidney ischemia and hypoxia. Similar results have been reported in patients with chronic kidney disease [32,33,34]. Interestingly, the R2* value of the renal medulla was correlated with the age of patients with AAV. Generally, an aging kidney is characterized by interstitial fibrosis, which is the final common pathway of chronic kidney diseases [37]. The association between the R2* value and age may be evidence of an association between ischemia and interstitial fibrosis.

## 5. Limitations

Our study had certain limitations. First, the present study applied DKI and BOLD MRI to detect kidney lesions in patients with AAV and found that some parameters of DKI and BOLD imaging have the potential to become noninvasive imaging markers for patients with AAV and kidney injury. However, this was a single-center study with a small sample size, and no adjustments were made for age or blood pressure. Moreover, the follow-up time of 6 months was short. Therefore, future studies are required to validate these findings.

## 6. Conclusions

In summary, significant differences in the imaging parameters of DKI and BOLD MRI were observed between patients with AAV and kidney injury and the healthy controls. The medullary MK value on DKI scanning may be a noninvasive biomarker for assessing the severity of kidney injury in patients with AAV.

## Figures and Tables

**Table 1:** Demographic and general clinical data of patients with AAV, and the imaging parameters of follow-up patients.

Parameters	Value
Total number of patients	20
Male/Female	8/12
Age (years)	55.8 ± 11.5
Height (cm)	159.5 ± 8.8
Weight (kg)	59.4 ± 7.6
Heart rate (bpm)	79.2 ± 10.1
Systolic pressure (mmHg)	133.8 ± 20.5
Diastolic pressure (mmHg)	77.5 ± 11.0
Edema of the face or lower limbs	10/20
Hemoptysis	4/20
Renal involvement	20/20
Pulmonary involvement	6/20
c-ANCA-positive	0/20
p-ANCA-positive	19/20
Hemoglobulin (g/L)	92.0 ± 19.9
White blood cell count (×10^9^/L)	10.0 ± 5.3
Platelet (×10^9^/L)	216.9 ± 101.3
Albumin (g/L)	31.6 ± 5.9
Globulin (g/L)	25.7 ± 9.1
Blood urea nitrogen (mmol/L)	17.8 ± 7.2
Creatinine (μmoI/L)	366.0 ± 203.9
Evaluated glomerular filtration rate (mL/min/1.73 m^2^)	15.41 (17.943)
Uric acid (μmol/L)	399.1 ± 103.1
Birmingham Vasculitis Activity Score	19.0 ± 4.1
Group reaching the end point of follow-up (9 patients)	
FA (cortex/medulla)	0.209 ± 0.064/0.228 ± 0.033
MK (cortex/medulla)	0.669 ± 0.202/0.663 ± 0.075
MD (cortex/medulla)	2.315 ± 0.437/2.44 ± 0.386
R2* (cortex/medulla)	27.278 ± 13.276/25.126 ± 6.449
Group not reaching the end point of follow-up (10 patients)	
FA (cortex/medulla)	0.182 ± 0.053/0.232 ± 0.054
MK (cortex/medulla)	0.580 ± 0.094/0.604 ± 0.057
MD (cortex/medulla)	2.514 ± 0.185/2.452 ± 0.207
R2* (cortex/medulla)	24.62 ± 8.813/30.85 ± 10.02

Mean ± SD (all such values); MK: mean kurtosis, MD: mean diffusivity, FA: fractional anisotropy; R2*: effective transverse relaxation rate; ANCA, antineutrophil cytoplasmic antibody.

**Table 2:** Correlation analysis of imaging parameters and clinical indexes in patients with AAV (r value).

Parameters	Renal Cortex				Renal Medulla			
	MK	MD	FA	R2*	MK	MD	FA	R2*
Age (year)	−0.004	0.078	0.015	0.242	0.05912	−0.117	0.181	0.469 *
Hemoglobin (g/L)	−0.502 *	0.238	0.337	0.199	−0.513 *	−0.023	0.311	0.043
BUN (mmol/L)	0.146	0.171	0.159	0.173	0.357	0.382	0.151	0.110
Creatinine (μmol/L)	0.328	0.179	0.002	0.113	0.591 **	0.419	0.077	0.084
e-GFR (mL/min/1.73 m^2^)	−0.363	−0.198	0.028	0.140	−0.539 *	−0.342	0.002	0.122
BVAS	0.408	−0.344	0.333	0.029	0.372	−0.186	0.124	0.157
Percentage of crescents	0.927 **	−0.630	0.062	0.488	0.741	−0.519	−0.395	0.488
Percentage of cellular crescents	−0.374	0.906 **	−0.394	0.317	0.197	0.512	−0.374	0.698
Percentage of cell fiber crescents	0.239	0.717	0.418	0.562	−0.120	0.837 *	−0.717	0.358
Percentage of fibrous crescents	0.374	−0.217	0.158	0.082	0.158	−0.906 **	−0.158	0.027
Percentage of tubular atrophy	0.126	0.456	0.141	0.072	−0.141	0.488	0.355	0.159
Percentage of interstitial fibrosis	0.126	0.456	0.141	0.072	−0.141	0.488	0.355	0.159

MK: mean kurtosis, MD: mean diffusivity, FA: fractional anisotropy; R2*: effective transverse relaxation rate; BUN, blood urea nitrogen; e-GFR, estimated glomerular filtration rate. * *p* < 0.05, ** *p* < 0.01.

**Table 3:** ROC analysis of diffusion parameters for the discrimination the severity of renal injury in AAV patients and the difference between AAV patients and the healthy controls.

Items	Cutoff Value	Sensitivity (%)	Specificity (%)	AUC
Discriminating AAV renal injury from HCs				
MK value of the renal cortex	>0.64	31.58	91.67	0.57
MK value of the renal medulla	>0.59	63.16	83.33	0.77
MD value of the renal cortex	<2.51	63.16	98.00	0.87
MD value of the renal medulla	<2.31	36.84	83.33	0.54
FA value of the renal cortex	<0.19	52.63	91.67	0.60
FA value of the renal medulla	<0.29	94.74	100.00	0.99
R2* value of the renal cortex	>15.42	90.00	93.33	0.95
R2* value of the renal medulla	<28.64	70.00	80.00	0.69
Discriminating AAV patients with Cr > 300 µmol from Cr < 300 µmol/L				
MK value of the renal cortex	>0.52	100.00	37.50	0.66
MK value of the renal medulla	>0.59	81.82	75.00	0.81
MD value of the renal cortex	>2.32	90.91	50.00	0.67
MD value of the renal medulla	>2.49	54.55	87.50	0.77
FA value of the renal cortex	<0.19	63.64	62.50	0.57
FA value of the renal medulla	<0.22	54.55	75.00	0.61
R2* value of the renal cortex	>29.29	33.33	87.50	0.55
R2* value of the renal medulla	<27.72	66.67	62.50	0.53

MK: mean kurtosis, MD: mean diffusivity, FA: fractional anisotropy; R2*: effective transverse relaxation rate; ROC, receiver operating characteristics; AUC: area under the curve, HCs: healthy controls; AAV, antineutrophil cytoplasmic antibody (ANCA)-associated vasculitis (AAV); Cr, creatinine.

## Data Availability

All relevant data are within the manuscript.
